# Stress Distribution of Pediatric Zirconia and Stainless Steel Crowns after Pulpotomy Procedure under Vertical Loading: A Patient-Specific Finite Element Analysis

**DOI:** 10.3390/jfb15090268

**Published:** 2024-09-14

**Authors:** Özgür Doğan

**Affiliations:** Department of Pediatric Dentistry, Faculty of Dentistry, Afyonkarahisar Health Sciences University, Afyonkarahisar 03030, Turkey; ozgurdogan1984@gmail.com

**Keywords:** pediatric zirconia crown, stainless steel crown, pulpotomy, mineral trioxide aggregate, finite element analysis

## Abstract

**Aim:** With modern dentistry advancements, children and parents have significantly raised aesthetic expectations in pediatric dentistry. Pediatric zirconia crowns (PZCs) provide a superior aesthetic appearance compared with stainless steel crowns (SSCs), making them a popular treatment option. However, a comparison of the compressive stresses caused by these crowns on the roots of primary teeth and alveolar bones has not been conducted. **Materials and Methods:** Cone beam computed tomography (CBCT) images of an eight-year-old female patient who experienced premature loss of a primary mandibular left second molar were obtained from a dental hospital database. Rhinoceros 4.0 software was used to process and simulate images. Under simulated chewing forces, stress on the PZC, SSC, and intact primary first molars as control groups, as well as their roots and alveolar bone structures, was assessed with finite element analysis. **Statistical Analyses:** Depending on whether the descriptive data were normally distributed, the Student t-test and Mann–Whitney U test were used. Quantitative variables differ between the two categories of qualitative variables. One-way ANOVA and Kruskal–Wallis H tests were used depending on standard distribution assumptions. *p* < 0.05 indicates statistical significance differences. **Results:** PZCs, SSCs, and cement layers were stressed according to von Mises values, while roots and alveolar bones were stressed according to maximum and minimum stress values. When assessing crowns, SSCs exhibited the highest von Mises stress values, followed by PZCs and control groups (*p* < 0.001). In the cement layer, SSCs obtained significantly higher values (*p* = 0.003). In the root area, minimum principal stress values are more critical. The highest values were obtained from the intact tooth, PZC, and SSC, respectively (*p* < 0.001). Alveolar bones did not differ significantly in minimum principal stress (*p* = 0.950). **Conclusions:** Restorative full-coverage crowns exhibited higher von Mises values than intact teeth, as per current research findings. The von Mises values were highest in SSC, while lowest in PZC. As a result of this condition, the cement layer and root areas had higher von Mises stress and compressive stress. Alveolar bones were not affected regardless of restoration type. PZC transmits higher stress due to its properties.

## 1. Introduction

Children and adults both have high aesthetic expectations in dentistry today [1]. Dental materials are advancing and are being validated as effective; dentists can provide children and their parents with information about these treatments so they to make an informed decision [2]. It has been over a decade since zirconia crowns have been used successfully in adults and in pediatric dentistry [3]. Zirconia for dental restoration in children (PZC) are popular full-coverage prefabricated aesthetic restorative materials that restore primary teeth, meeting aesthetic expectations and functional requirements in pediatric patients. However, further research is needed to enhance their efficacy [4].

Zirconia ceramic steel, also referred to as zirconia, is a crystalline dioxide of zirconium [5]. In addition to their aesthetic qualities, zirconia crowns are also three times stronger than traditional porcelain-fused metal crowns because they are milled from a single-sintered crystal block [6]. Zirconia crowns have a rigid structure that prevents them from bending or recontouring during treatment, enhancing compatibility with the tooth. Passive seating is recommended by manufacturers, which weakens the tooth further after more preparation is made from PZC’s primary teeth. As a result of this procedural requirement, pulpal exposure is more likely in primary teeth and always requires a pulpotomy or pulpectomy procedure [3].

Finite element analysis (FEA), initially used in engineering, is now widely used in determining stress and strain on dental restorative materials, teeth, and supporting bone structures [7,8,9,10]. FEA begins with transforming the model’s geometry into subdivisions consisting of finite elements and attaching nodes to each subdivision to create a mesh structure. With modeling and simulation programs, complex models can be constructed more affordably than real models, whose stresses and strains can be precisely, clearly, accurately, and safely calculated, taking into account the mechanical properties of these materials. While FEA is useful for orthodontics, endodontics, implant surgery, and prosthetic treatments, its application in pediatric dentistry remains limited [7].

Stainless steel crowns (SSCs) are restorative materials in pediatric dentistry. Typically, they are used to treat atypical and large cavities caused by early childhood caries. Following pulpotomies and pulpectomies, SSCs help maintain crown integrity and prevent microleakage [11]. It is the most preferred type of restorative crown in pediatric dentistry because it is cost-effective, durable, and requires minimal technical sensitivity during application. However, due to its aesthetic appearance, its acceptability decreases daily for both children and parents [12].

For both primary anterior and posterior teeth, PZCs provide high aesthetic satisfaction [13]. By their highly marginal biocompatibility and low dental plaque accumulation, PZCs prevent SSC-negative properties by causing lesser gingival irritation [14]. Preformed pediatric crowns are recommended by the American and British Association of Pediatric Dentistry guidelines for treating primary molars with extensive caries on two and three surfaces. Using these crowns after pulpotomy and pulpectomy is suggested [15,16].

Studies on internal and external root resorptions and their causes are ongoing, particularly after pulpotomy and pulpectomy [17,18,19,20,21]. While internal resorption may be caused by the histopathological condition of pulps and pulp-covering materials (e.g., mineral trioxide aggregate, calcium hydroxide) [17,18], external resorption remains unclear [19,20,21].

PZCs are rapidly becoming the leading alternative to SSCs. No research has been conducted on the stresses and strain values created by full-coverage crowns used for restorative purposes on primary molars restored with SSCs and PZCs. The situation raises a big question: when determining the type of crown for restoration, pediatric dentists first consider how the restored tooth will react to the stress caused by chewing forces. They create a challenge by balancing material loss, parental desire, and price. This study aims to determine the amount, intensity, and location of stress on intact tooth, SSC, and PZC crowns caused by chewing forces on the crown, root, and bone structures. This study hypothesizes that intact teeth and teeth restored with SSCs or PZCs after pulpotomy will demonstrate equal stress and strain values in the crowns, cement layer, roots, cortical, and cancellous bones.

## 2. Materials and Methods

An assessment of the restorative treatment of a primary first tooth after pulpotomy with SSC and PZC was conducted using clinical scenario simulation. To ensure accurate FEA results, study models were meticulously constructed to simulate clinical conditions precisely. In this study, an eight-year-old female patient was selected from the cone beam computerized tomography (CBCT) database at Afyonkarahisar Health Sciences University research hospital after approval from the local ethics committee (2024/179). Informed consent was obtained from the patient, as well as parental permission. A 3D geometric model was constructed from CBCT volumetric data of the orofacial region with a 0.2 mm section thickness. DICOM data were exported to 3D Doctor Version 4.0 Software (Able Software Corp., Lexington, MA, USA) for segmentation in the Digital Imaging and Communications in Medicine 3.0 format. Using an interactive segmentation method based on Hounsfield units, the cancellous and cortical bones were segmented using 3D Doctor Software (Table 1).

**Figure 1:**
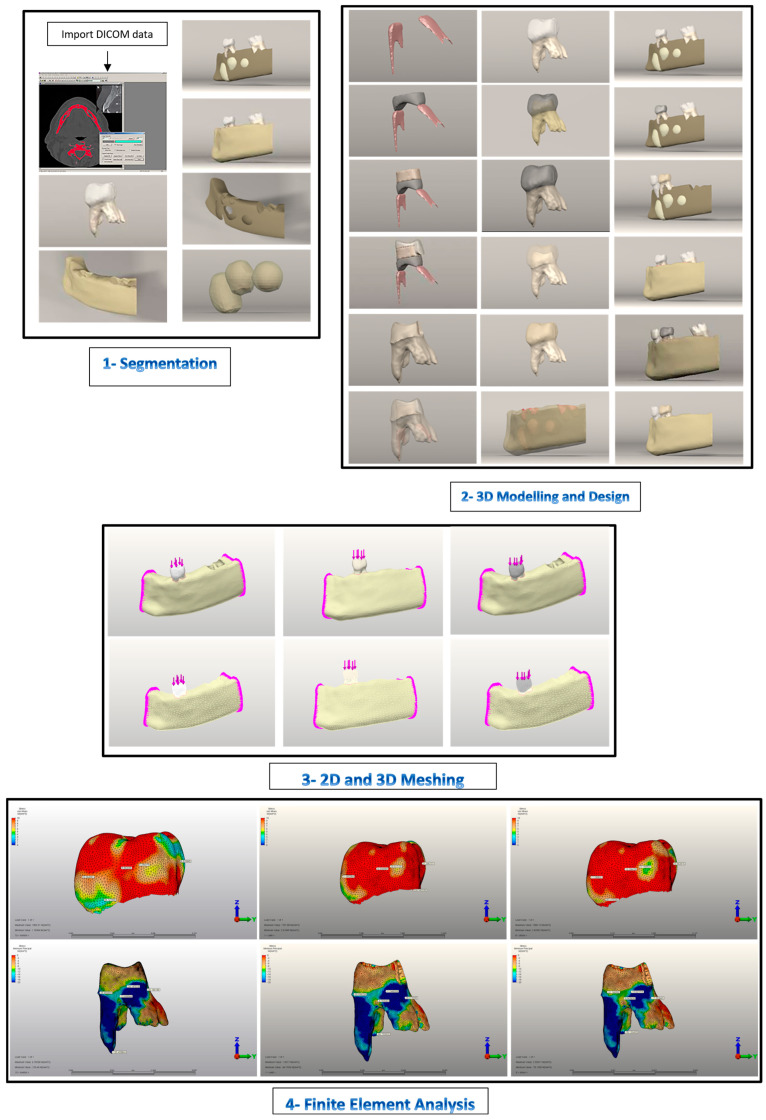
1: CBCT dataset imported into 3D Doctor software for segmentation of teeth, cortical, and cancellous bones. 2: Assembly of teeth with pulps, cortical and cancellous bones, and fixed space maintainers using Rhinoceros software. 3: Assembled study models were exported from Rhinoceros software to VRMesh for meshing and generating simulation models by VRMesh Studio. 4: All models were stress-analyzed using Algor Fempro Vers 23.0 software. Some subfigures are reprinted from Ref. [7].

After automated segmentation, the geometric data were manually revised and corrected to smooth the model and fill in any gaps. Using the 3D complex rendering method, the 3D models were processed and saved as stereolithography files (.stl). Tooth-bone/crown (TB-CR) surface geometries include enamel, dentin, alveolar bone, roots, periodontal ligaments (PDLs), gingiva, and root canals. The TB-CR model included tooth 74, a primary mandibular first molar that underwent pulpotomy. It was fitted with an intact enamel, stainless steel crown (SSC), and zirconia for dental restorations in children (PZC) (Figure 1). Rhinoceros 4.0 software (3670 Woodland Park Ave N, Seattle, WA 98103, USA) was used to import and assemble all 3D spatial coordinates. The cortical and cancellous bones, teeth, and PDL were then reconstructed based on their anatomical dimensions. Three patterned crowns were also designed using Rhinoceros 4.0 software 3670 Woodland Park Ave N, Seattle, WA 98103, USA) Using a Boolean operation to surround tooth 74’s crown, 0.2 mm was assigned to the PDL thickness [7,8] (Figure 1).

To capture the dimensional and topographic details of enamel, dentin structures, and three crowns, a 3D mesh was created using VR Mesh Studio (VirtualGrid Inc., Bellevue, WA, USA) [22,23]. Three-dimensional FEA was conducted using the subject-specific geometry of the TB-CRs. In the meshing process, TB-CRs were created as quadratic tetrahedron elements using a surface model approach [7]. This approach aimed to create a more accurate and realistic 3D model by incorporating more intricate details. To improve mesh quality, the models were subjected to convergence analysis. The mesh convergence analysis using h-adaptivity determined that all models achieved convergence once the inter-analysis stress variation was below 3% [7]. There was a decreased average of 147.610 nodes and 672,926 elements, with a calculated maximum error of 0.90% (Table 2). A 0.1 mm element size was used for quality, especially between the CRs-TB-PDL complexes. This modeling technique generated a high-quality mesh structure with the maximum number of nodal elements for stress analysis [24]. An adaptive meshing option that preserves enamel and dentin thin and fine structures was also used [8,25]. Three TB-CR complexes were refined before being transferred to Algor Fempro Vers. 23.0 software (ALGOR, Inc. 150 Beta Drive Pittsburgh, PA 15238-2932, USA) for FEA. Three-dimensional coordinates of the models were preserved during this process (Figure 1) [22].

Young modulus and Poisson’s ratios were used to define the TB-CRs components’ material physical properties. The models incorporate the behavior of all dental tissues and materials. Using Algor Fempro software, material properties were defined according to previous FEA studies (Table 3). All dental tissues and materials were assumed to be homogeneous, isotropic, and linearly elastic (Figure 1).

### 2.1. Model Generation

**Figure 2:**
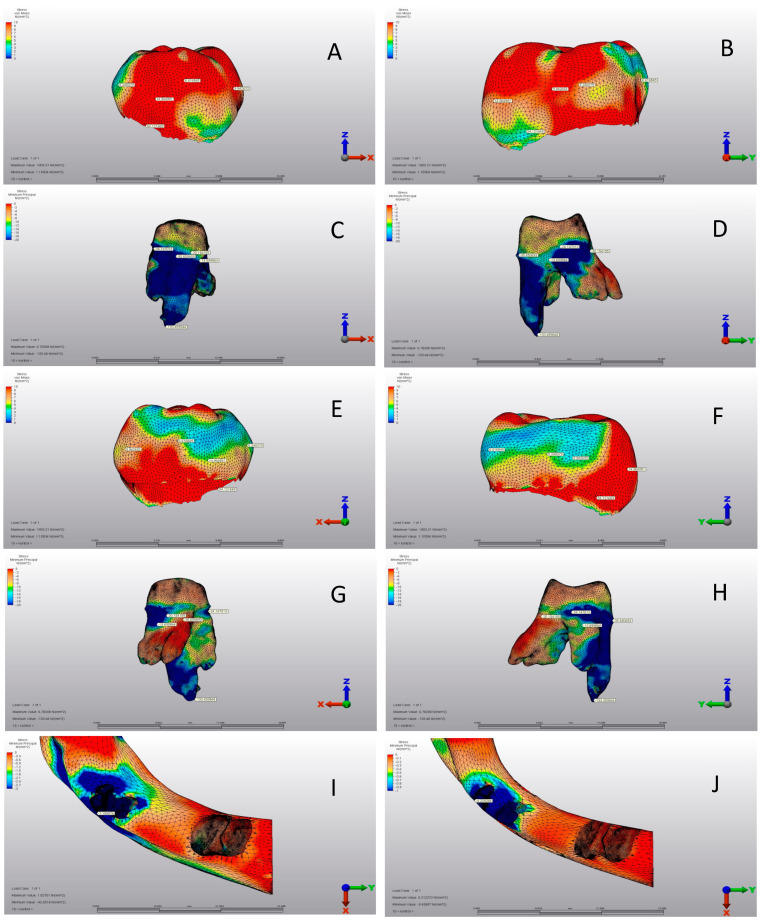
Control group. (**A**) Mesial view of the crown (intact enamel). (**B**) Buccal view of the crown. (**C**) Mesial view of the root structure. (**D**) Buccal view of the root structure. (**E**) Distal view of the crown. (**F**) Lingual view of the crown. (**G**) Mesial view of the root structure. (**H**) Lingual view of the root structure. (**I**) Cortical bone structure. (**J**) Cancellous bone structure.

***Control Model:*** This model represents the chewing force applied to a healthy, intact tooth (Figure 1 and Figure 2A–J). This model was taken directly from the CT database. Contact teeth were excluded from boundary conditions to study the analysis results on a single tooth.

**Figure 3:**
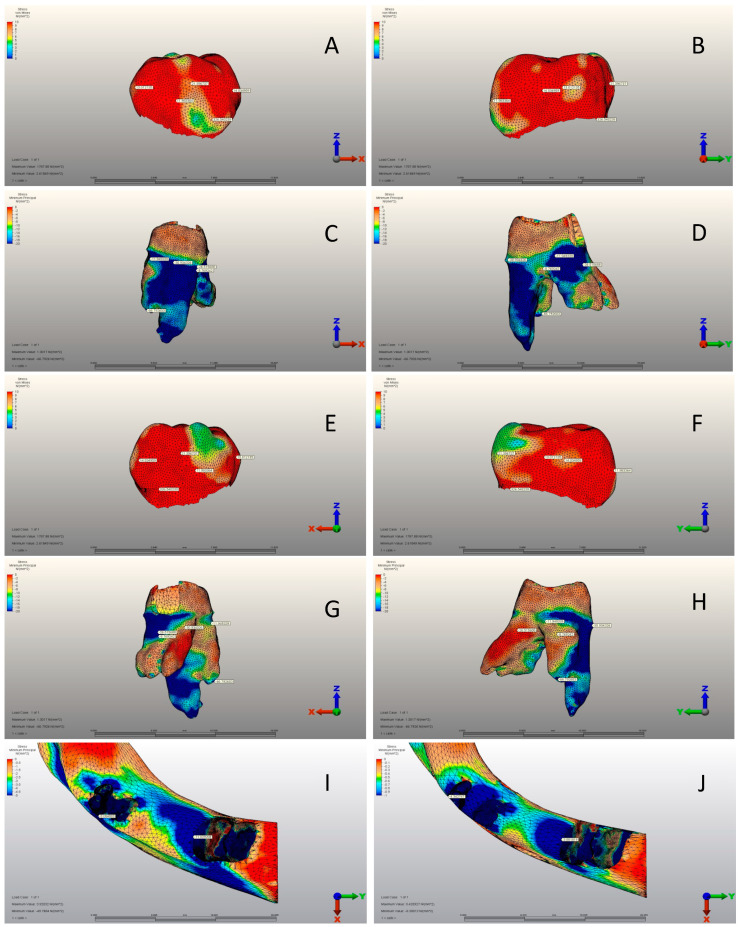
SSC group. (**A**) Mesial view of the crown. (**B**) Buccal view of the crown. (**C**) Mesial view of the root structure. (**D**) Buccal view of the root structure. (**E**) Distal view of the crown. (**F**) Lingual view of the crown. (**G**) Mesial view of the root structure. (**H**) Lingual view of the root structure. (**I**) Cortical bone structure. (**J**) Cancellous bone structure.

***Stainless Steel Crown Model:*** This model represents a scenario where an SSC is positioned on tooth 74 (Figure 1 and Figure 3A–J). NuSmile^®^ pre-contoured SSC instruction was followed for generating a prepared tooth model. The intact tooth model’s enamel layer was initially reduced by 1.5 mm occlusally. Next, the entire tooth tissue was thinned all around, and a 1.5 mm knife-edge cut was made below the gum line to prepare the tooth model for SSC cementation, leaving 30% dentin thickness [31,32]. This model generation is preferred to achieve standardization by achieving the same level of reduction as the zirconia crown preparation, despite the Instructions for Use stating that buccal and lingual cutting is unnecessary.

**Figure 4:**
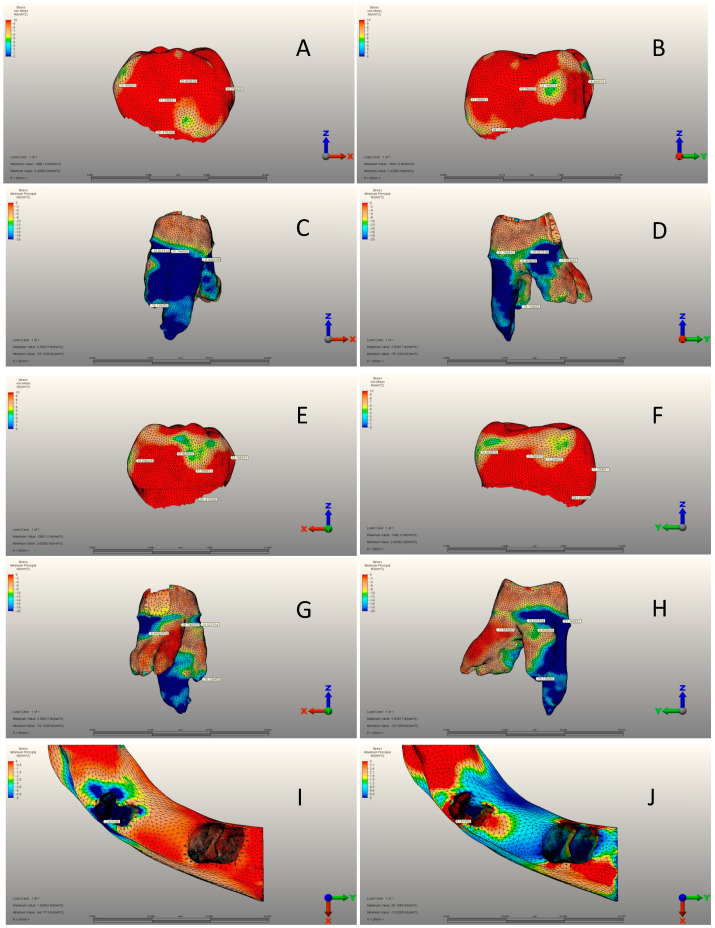
PZC group. (**A**) Mesial view of the crown. (**B**) Buccal view of the crown. (**C**) Mesial view of the root structure. (**D**) Buccal view of the root structure. (**E**) Distal view of the crown. (**F**) Lingual view of the crown. (**G**) Mesial view of the root structure. (**H**) Lingual view of the root structure. (**I**) Cortical bone structure. (**J**) Cancellous bone structure.

***Pediatric Zirconia Crown Model:*** This model represents a scenario where a PZC is positioned on tooth 74 (Figure 1 and Figure 4A–J). In the preparation of the tooth model, a 1.5 mm reduction was made from the occlusal surface in accordance with the NuSmile^®^ Zirconia Instruction for Use. The tooth was then circumferentially reduced by 1.5 mm. Cutting the subgingival 1.5 mm knife edge prepared the entire tooth for PZC cementation [32,33]. By exceeding the Instructions for Use by 0.25 mm, tooth reduction procedures in both models were equalized.

#### 2.1.1. Boundary Conditions

The FEA applied the following boundary conditions: The coronal, sagittal, and transverse planes of the mandible were fixed. The crown-cutting plane was constrained out-of-plane. Cortical and cancellous bones were modeled as continuous and inseparable. Simulations were performed using a bonded contact algorithm that only allowed small sliding movements between teeth and the periodontal ligament (PDL). In the TB-CR complex, a displacement field was applied between the bonded TB, CR, and CR–cement layer (0.02 mm) contacts [28,34,35]. A friction coefficient of 0.2 was set between the tooth and CRs [35,36].

The connection between the CR-TB complex was represented as a surface-to-surface contact condition. The CRs were not allowed to separate from the tooth complex during vertical chewing force loading [29,37].

#### 2.1.2. Simulation of Force Loading and FEA

Under static loading conditions, a 289.28 N force was exerted vertically on the occlusal surface of tooth 74 along its long axis for one second. The procedure is similar to previous studies [23,38,39]. During vertical chewing force stimulation, a total force of 289.28 N was evenly distributed over five different functional contact points (Figure 1). It is reasonable to examine the overall stress distribution in the TB-CR complex using point loading conditions, despite the minimal loading area that would be present in an actual clinical situation. We used Algor Fempro software to analyze stress distribution through stress analysis [22]. The stress points to emphasize were determined during the selection process based on an evaluation of the PZCs group, which was the primary focus of our research. After identifying the pinpoints within the PZCs group, these points were adapted with those of other groups to ensure standardization of numerical comparisons across all groups. Then, the von Mises and minimum principal values were presented only for the selected pinpoint in that area.

#### 2.1.3. Statistical Analyses and Interpretation of Analysis Results

The data obtained from the present study were analyzed using the Statistical Package for the Social Sciences (SPSS) software (version 20.0). Descriptive statistics were used to analyze the data. Quantitative variables were expressed as mean ± standard deviation and median (minimum–maximum), while qualitative variables were expressed as the number of patients (percentage, %). Depending on whether standard distribution assumptions were met, two statistical tests were used if a qualitative variable was different from a quantitative variable. The Student-t test was used if the assumptions were met, and the Mann–Whitney U test if they were not. The one-way ANOVA test was used to determine if there were differences between categories of a qualitative variable with more than two categories if normal distribution assumptions were met. If the assumptions were not met, we used the Kruskal–Wallis H test. This analysis was conducted in terms of a quantitative variable (*p* < 0.05 indicates a statistically significant difference).

The SSC and PZC stress values were calculated using von Mises stresses. On tooth 74, the buccal, lingual, mesial, and distal root surfaces were measured for maximum and minimum principal stresses. Additionally, the highest maximum and minimum principal stresses of root structures and cortical and cancellous bones were calculated due to ductile characteristics. We calculated the von Mises equivalent stress values for brittle structures of PZC, SSC, and cement layers [23]. Both types of stress were visualized using a linear color scale. The von Mises and maximum principal stresses were highlighted in red in areas with the highest stress concentration. In contrast, areas with the lowest minimum principal stresses were displayed in blue. The principal stress values were also examined, with positive values indicating tensile stresses (maximum principal) and negative values indicating compressive stresses (minimum principal). This study highlights the tooth area experiencing the highest stress during chewing. It is therefore more important to consider minimum principal stress values.

## 3. Results

### 3.1. Pediatric Zirconia, Stainless Steel, and Control Group Crowns

There was a significant difference between the three groups in terms of all variables (*p* < 0.001) (Table 4). The mean mesial view was 5.24 ± 2.01 MPa, 13.01 ± 4.02 MPa, and 13.76 ± 3.56 MPa in the CG, SSC, and PZC groups, respectively. PZC had the highest average buccal view, whereas SSC had the lowest. While the mean distal view was 9.94 ± 1.92 MPa, 14.03 ± 2.79 MPa, and 13.23 ± 2.46 MPa in the CG, SSC, and PZC groups, respectively. SSC had the highest averages for the lingual view and von Mises stress variables, while CG had the lowest (Table 4) (Figure 2A–J, Figure 3A–J and Figure 4A–J).

### 3.2. Pediatric Zirconia, Stainless Steel, and Control Group Roots

According to Table 5, there were significant differences among groups for all variables except for mesial view minimum principal stress (*p* < 0.001). The mean of mesial view maximum principal stress was 0.89 ± 0.08 MPa, 0.21 ± 0.06 MPa, and 0.05 ± 0.01 MPa in the CG, SSC and PZC groups, respectively. SSC had the highest mean buccal view maximum principal stress, while PZC had the lowest. CG had the highest mean buccal view minimum principal stress, while SSC had the lowest. Compared to CG, the mean distal view maximum principal stress was 0.25 ± 0.06 MPa. SSC had a mean of 0.90 ± 0.16 MPa, while the PZC group had a mean of 0.08 ± 0.02 MPa. PZC had the lowest mean distal view minimum principal stress, whereas SSC had the highest. Also, CG had the highest averages of lingual view maximum and minimum principal stress and the highest principal stress. In contrast, SSC had the lowest averages (Table 5) (Figure 2A–J, Figure 3A–J and Figure 4A–J).

### 3.3. Pediatric Zirconia, Stainless Steel, and Control Group Cement Layers

**Figure 5:**
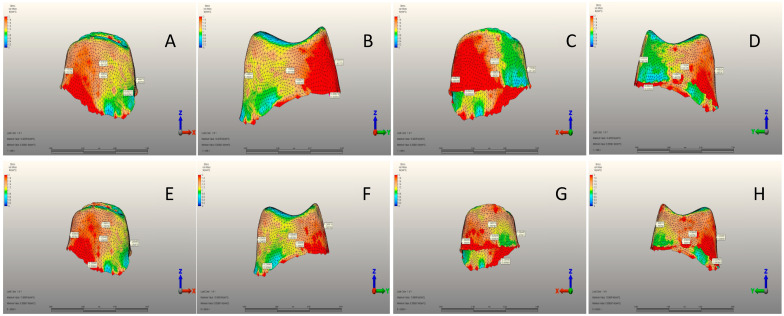
(**A**) Mesial view of the cement layer of the SSC group. (**B**) Buccal view of the cement layer of the SSC group. (**C**) Distal view of the cement layer of the SSC group. (**D**) Lingual view of the cement layer of the SSC group. (**E**) Mesial view of the cement layer of the PZC group. (**F**) Buccal view of the cement layer of the PZC group. (**G**) Distal view of the cement layer of the PZC group. (**H**) Lingual view of the cement layer of the SSC group.

Table 6 compares all variables. Significant differences were observed between the mesial view, lingual view, and max. von Mises stress (*p*: 0.007, 0.027, and 0.003, respectively). The mean of the mesial view was 1.23 ± 0.25 MPa in SSC and 1.49 ± 0.32 MPa in PZC. PZC had a significantly higher mean for the lingual view. While the mean max. von Mises stress was 16.61 ± 3.64 MPa in SSC and 13.58 ± 2.01 MPa in PZC (Table 6) (Figure 5A–H).

Table 7 shows no significant differences among the three groups for any variable (*p* > 0.05). The mean of cortical bones’ maximum principal stress was 9.02 ± 1.85 MPa, 9.74 ± 2.11 MPa, and 9.16 ± 1.99 MPa in the CG, SSC and PZC groups, respectively. SSC had the highest average for cortical bones’ minimum principal stress, whereas CG had the lowest. The mean cancellous bone maximum principal stress was 1.22 ± 0.20 MPa, 1.24 ± 0.22 MPa, and 1.30 ± 0.26 MPa in the CG, SSC and PZC groups, respectively. SSC had the highest average for cancellous bones’ minimum principal stress, while PZC had the lowest (Table 7) (Figure 2I,J, Figure 3I,J and Figure 4I,J).

## 4. Discussion

This study is the first to evaluate compressive stresses on teeth caused by SSCs and PZCs. This study examines the resistance of teeth to occlusal-centric chewing forces, in addition to the ways in which these forces are transmitted to the roots and alveolar bone. Intact enamel tissue transmits higher amounts of chewing force to the roots of the underlying bone structure. This means it remains more rigid against chewing forces and does not absorb them but instead transmits them to the underlying tissues. In this study, the initial null hypothesis was rejected. It is evident from the results that SSCs absorb high levels of von Mises stress by stretching against chewing forces [26,28,40]. PZC crowns accumulate fewer Von Mises values than SSC crowns.

Both SSC and PZC crowns accumulated lower von Mises stress values than intact enamel tissue in the root area due to the cement layer underneath some of the principal stress values accumulating. Due to the flexible structure of the crowns, the SSCs transmitted more compressive stress in the PZC root area along with the crown stress values. In all groups, compressive stress values were nearly equally transmitted to cortical and cancellous bones. In part, this is due to the periodontal ligament’s buffering capacity against chewing forces [7,8].

Experimental simulations clearly indicate that the material’s physical properties play a role in determining the compressive stress values on the crown. The different compressive stress values observed around the roots are related to the glass ionomer cement used in luting [26,28,29,40]. Consequently, intact enamel tissue in the root area of the control group was found to have higher compressive stress values.

In dentistry, FEA has been a valuable tool for many years. Chewing forces are used to study the stress characteristics of restorative and prosthetic materials [7,23]. FEA is an engineering tool used to analyze dental restorative materials’ properties and design characteristics while in use, without human ethical considerations [27,40]. By simulating various scenarios, FEA enables researchers to conduct numerous experiments without involving human, clinical, and laboratory experiments [8]. By understanding the characteristic features of dental restorative materials under chewing forces, we can increase the lifespan of both the applied tooth and the restorative material used. Their harmful effects on the tissues can also be prevented [40,41].

This study aimed to determine the magnitude and location of stresses caused by PZC and SSC on primary molar teeth when subjected to vertical chewing force loading. It marked the first such analysis in the literature. During our study, we restored an 8-year-old female patient’s mandibular right primary first molar tooth using MTA + GIC + composite resin following a pulpotomy. We prepared the crown, cemented the PZC and SSCs using RMCIS, and applied maximum vertical chewing force as possible in mixed dentition. The amount and localization of tensile and compressive stresses accumulated in the full-coverage crown, cement layer, crown, roots, and cortical–cancellous bones were evaluated.

For a biological seal and success, SSC is crucial after pulpal treatment [42]. By mimicking the anatomical form of teeth, they restore function to decayed primary teeth [43]. SSCs are available in several sizes for first and second molars. For an optimal fit, they can be trimmed, crimped, and reshaped. Due to its convexity, the crown margins are retained by the primary molar teeth undercutting the cervical area [11].

Resin-modified glass ionomer cement (RMGI) was used as a luting agent in this study. A debate continues over the best luting agent for SSCs and PZCs crowns [11,29]. Despite trimming and crimping, SSCs can improve retention; however, these processes cannot be applied to PZCs. Hence, luting cement needs both retention and sealing properties. SSC crowns bonded successfully with RMGI over conventional glass-ionomer cement and polycarboxylate cement [11,44]. Previous research suggests using RMGI as a luting agent when optimal moisture control is essential [29]. When dealing with behavioral and time-related issues, traditional GIC is recommended [11]. Since our study was an experimental simulation, RMGI was the most appropriate luting cement. In both groups, the same type of cement was used, so the results were not affected by the luting agent.

RMCI cement thickness was 200 µm in this study. For zirconia crowns, cement thicknesses ranging from 20 to 300 µm do not significantly affect fracture resistance [28,29]. According to Guler et al., the cement thickness was not tested in their study; instead, they chose a thickness of 300 μm. RMGI cement types accumulate more von Mises stress than conventional GIC [28]. Prabhakar et al. examined cement with a 200 µm thickness in a study. The amount of material lost from the tooth exceeded the cement thickness, impacting its ability to withstand chewing forces [26]. Waly et al.’s study examined the cement types used in the cementation of SSC crowns and found that the stiffness character of the cementation material is more important than its thickness. Consequently, stiffer cements reduce stress accumulation on teeth and restorative materials. Chung et al. reported a decrease in von Mises values in PZC RMGIC crowns when testing cement thicknesses of 100–500–1000 µm. With increasing preparation in the tooth, the amount of stress accumulated on the cement thickness increased. They reported no significant difference in stress levels between 100 and 500 µm cement thickness but substantial increases at 1000 µm thickness, particularly under oblique loading. The studies show that using a 200 μm cement layer in our study aligns with previous research. As a result, cement thickness is not a concern when studying the stress on restorative crowns, dental crowns, roots, and bone structures.

Previous research has demonstrated that muscular and neuromuscular development increases masticatory forces. The most significant increase occurs during the transition from early to late mixed dentition [23,39]. For finite element applications in biomechanics, there is an ongoing debate regarding the selection and application of maximum chewing force in primary and mixed dentition [23,28,39]. Studies have shown that maximum von Mises stresses are distributed differently in FEA when applied force directions and application areas vary [26,28]. Many studies have indicated that the maximum bite force in primary and mixed dentition ranges from 161 to 330 N [28,39,45,46]. This study involved 8-year-olds at the mixed dentition stage, from whom test models were obtained. Using the calculation technique of Owais et al., we determined that the maximum bite force for an 8-year-old child is 289.23 N [23,39]. Despite using this calculation technique, the maximum bite force applied could have been either 245 N [46] or 330 N [28,40], as used in previous studies. However, this would only alter the amount of force, not the location of the resulting stress. Eccentric chewing forces may have influenced the results. This study aimed to present the results clearly. It acknowledged that eccentric chewing forces may be difficult to standardize, making interpretation challenging. Nevertheless, this study may serve as a foundation for future research.

Compared to other research, this study has specific limitations and notable differences. Pediatric crowns are most commonly used to treat decay in male and female mandibular primary second molars [40]. One limitation of this study is that it focused on the first molar of the primary mandibular. The simulation in our study was based on a working model obtained from the hospital database. In light of ethical concerns, an additional CBCT from another patient was not deemed appropriate [47]. So, further studies involving primary mandibular second molars are necessary. Tests were conducted on simulation models created by scanning extracted teeth with a laser scanner [40,48]. Despite this, the researchers encountered challenges in the simulation models when creating the cortical and cancellous bones [29,40,48]. Tests on intact tooth models were also conducted. This study created a completely routine clinical scenario. It is possible to develop a variety of scenarios and experiment with this subject in further FEA studies. Our study involved creating a model of a decayed tooth and restoring it with a full-coverage crown. This pattern may have influenced the amount and location of stress experienced. Cementation surfaces can be increased during pulpotomy procedures to enhance full-coverage crown retention. This can be carried out by finishing the restorations with open pulpal access after applying the base material for the pulpotomy and then cementing the crowns [49]. There are several factors that influence the minimum principal stress, including the type of luting cement, the preparation method, the occlusal convergence angles (taper), and the remaining clinical crown height [12]. A more detailed analysis of the localization of compressive and tensile stresses should be further conducted with variations in these parameters. Future studies should evaluate whether internal and external resorptions observed after pulpotomy occur in areas with higher stress accumulation in the root area of primary teeth with PZC restorations. In this study, simulations were conducted assuming that all intact teeth, SSC, and PZCs have the same morphology. However, the current situation is limited. The occlusal geometry is considered the most important of these surfaces. Different prefabricated crown brands exhibit distinct occlusal morphologies. Conducting a study on brand-specific occlusal morphology would make the results more challenging. The validity of our results will be improved by incorporating this issue into future studies.

## 5. Conclusions

When vertical chewing force is applied, primary dentition experiences the greatest force. The results were evaluated within this study’s limitations.

1. The von Mises stress values for SSCs are higher regardless of the cement used. Steel material’s flexibility and force-absorbing properties are likely to be responsible for this situation.
2. Compressive stress values were highest in intact tooth root areas. Without intervention in the stress transmission mechanism, the intact tooth’s root area experiences higher stress values. This mechanism involves the use of restorative materials and cement. Consequently, they alter the stress transmission mechanism, causing stress to accumulate on them.
3. In PZC crowns, the zirconia material’s rigidity leads to a higher transmission of von Mises stresses to the root area.
4. Despite the same cement layer thickness in PZC and SSC crowns, SSC crowns achieved higher compressive values. It is believed that steel absorbs chewing stress, distributes it throughout the crown, and transmits it to the cement layer. Due to its rigid structure, zirconia seems to have a linear stress transmission mechanism in PZCs.
5. In the mixed dentition, cortical and cancellous bones are not sensitive to chewing forces transmitted through crowns in full-coverage crown restorations. Cement or crown composition had no effect on this situation. Possibly, this is due to the periodontal ligament’s buffering capacity, which absorbs chewing force.

## Figures and Tables

**Table 1:** Hounsfield units for simulation groups.

Part	Hounsfield Unit	
	Min	Max
**Cortical bone**	662	1988
**Cancellous bone**	148	661
**Enamel**	1553	2850
**Dentin**	1200	1552

**Table 2:** Nodes and element numbers for working groups.

Groups	Number of Nodes	Number of Elements
Control	129,863	600,043
Pediatric zirconia group	156,290	708,986
Stainless steel group	156,678	709,751

**Table 3:** Anatomical structures and materials’ elastic modulus and Poisson’s ratios for FEA.

Materials	Elastic Modulus (MPa)	Poisson’s Ratio
Primary teeth enamel	80,349	0.33 [26]
Primary teeth dentine	19,890	0.31 [26]
Pulp	30	0.45 [8]
Periodontal ligament	50	0.49 [27]
Stainless steel crown	200,000	0.33 [28]
Pediatric zirconia crown	205,000	0.19 [29]
Mineral trioxide aggregate	11,700	0.31 [30]
Cortical bone	13,700	0.30 [8]
Cancellous bone	1400	0.30 [8]
Resin-modified glass ionomer cement	3700	0.30 [28]
Glass ionomer cement	10,800	0.25 [30]

**Table 4:** Comparing variables among groups.

Crown Variables		Control Group	Stainless Steel Crown	Pediatric Zirconia Crown	*p*-Value
Mesial view (MPa)	Mean ± SD	5.24 ± 2.01	13.01 ± 4.02	13.76 ± 3.56	**<0.001 ^a^**
	Median (min–max)	4.88 (2.09–9.05)	13.17 (5.95–19.13)	13.75 (8.39–22.18)	
Buccal view (MPa)	Mean ± SD	14.96 ± 2.91	11.98 ± 3.05	17.29 ± 2.73	**<0.001 ^a^**
	Median (min–max)	15.98 (7.57–20.87)	12.64 (5.82–17.15)	17.82 (12.38–22.82)	
Distal view (MPa)	Mean ± SD	9.94 ± 1.92	14.03 ± 2.79	13.23 ± 2.46	**<0.001 ^a^**
	Median (min–max)	9.97 (6.57–13.34)	13.74 (10.88–21.10)	13.37 (9.84–17.63)	
Lingual view (MPa)	Mean ± SD	6.61 ± 1.76	21.39 ± 4.06	15.96 ± 2.97	**<0.001 ^a^**
	Median (min–max)	6.71 (4.33–9.53)	22.06 (14.50–27.45)	17.02 (10.78–20.41)	
Max. von Mises stress (MPa)	Mean ± SD	54.12 ± 10.98	326.54 ± 24. 84	281.47 ± 27.76	**<0.001 ^a^**
	Median (min–max)	54.22 (34.47–73.13)	330.10 (271.50–359.29)	280.26 (238.81–317.58)	

SD: standard deviation, Min: minimum, Max: maximum, ^a^: one-way ANOVA test.

**Table 5:** Comparing root structure variables among groups.

Root Structure Variables			Control Group	Stainless Steel Crown	Pediatric Zirconia Crown	*p*-Value
Mesial view (MPa)	Maximum principal stresses	Mean ± SD	0.89 ± 0.08	0.21 ± 0.06	0.05 ± 0.01	**<0.001 ^a^**
		Median (min–max)	0.89 (0.78–1.13)	0.21 (0.11–0.30)	0.05 (0.03–0.07)	
	Minimum principal stresses	Mean ± SD	35.65 ± 6.92	30.93 ± 6.25	31.74 ± 6.24	0.056 ^a^
		Median (min–max)	34.29 (26.26–48.97)	31.22 (17.37–40.83)	30.53 (22.38–42.61)	
Buccal view (MPa)	Maximum principal stresses	Mean ± SD	0.06 ± 0.02	0.12 ± 0.03	0.01 ± 0.01	**<0.001 ^b^**
		Median (min–max)	0.06 (0.03–0.11)	0.12 (0.07–0.18)	0.01 (0.00–0.02)	
	Minimum principal stresses	Mean ± SD	13.69 ± 2.30	8.76 ± 2.96	8.95 ± 2.00	**<0.001 ^a^**
		Median (min–max)	13.38 (9.70–17.47)	9.19 (4.31–13.75)	8.48 (4.69–12.96)	
Distal view (MPa)	Maximum principal stresses	Mean ± SD	0.25 ± 0.06	0.90 ± 0.16	0.08 ± 0.02	**<0.001 ^a^**
		Median (min–max)	0.25 (0.13–0.37)	0.92 (0.53–1.13)	0.08 (0.05–0.13)	
	Minimum principal stresses	Mean ± SD	20.15 ± 4.20	26.51 ± 3.61	17.55 ± 3.58	**<0.001 ^b^**
		Median (min–max)	19.52 (13.86–26.80)	27.49 (20.13–31.69)	17.58 (11.94–25.27)	
Lingual view (MPa)	Maximum principal stresses	Mean ± SD	1.92 ± 0.33	0.42 ± 0.13	0.45 ± 0.09	**<0.001 ^a^**
		Median (min–max)	1.91 (1.42–2.64)	0.45 (0.21–0.59)	0.47 (0.28–0.61)	
	Minimum principal stresses	Mean ± SD	24.15 ± 4.63	11.94 ± 2.73	15.02 ± 2.88	**<0.001 ^a^**
		Median (min–max)	24.77 (14.35–32.52)	12.03 (5.54–17.51)	15.29 (8.41–21.90)	
The highest principal stress (MPa)	Maximum principal stresses	Mean ± SD	53.71 ± 12.20	37.24 ± 9.36	43.48 ± 9.20	**<0.001 ^a^**
		Median (min–max)	50.58 (35.64–75.60)	37.65 (17.78–55.69)	41.23 (26.16–57.78)	
	Minimum principal stresses	Mean ± SD	133.44 ± 19.67	66.79 ± 11.39	78.12 ± 11.98	**<0.001 ^a^**
		Median (min–max)	130.83 (101.20–174.26)	65.08 (41.15–91.45)	78.41 (59.45–95.38)	

SD: standard deviation, Min: minimum, Max: maximum, ^a^: one-way ANOVA test, ^b^: Kruskal–Wallis H test.

**Table 6:** Comparing cement layer variables among groups.

Cement Layer		Stainless Steel Crown	Pediatric Zirconia Crown	*p*-Value
Mesial view (MPa)	Mean ± SD	1.23 ± 0.25	1.49 ± 0.32	**0.007 ^a^**
	Median (min–max)	1.24 (0.86–1.62)	1.46 (0.79–2.13)	
Buccal view (MPa)	Mean ± SD	1.45 ± 0.43	1.63 ± 0.58	0.270 ^a^
	Median (min–max)	1.51 (0.69–2.11)	1.65 (0.83–2.93)	
Distal view (MPa)	Mean ± SD	1.46 ± 0.37	1.29 ± 0.28	0.110 ^a^
	Median (min–max)	1.46 (0.56–2.11)	1.35 (0.70–1.79)	
Lingual view (MPa)	Mean ± SD	1.56 ± 0.40	1.29 ± 0.34	**0.027 ^a^**
	Median (min–max)	1.61 (0.87–2.22)	1.38 (0.63–1.87)	
Max. von Mises stress (MPa)	Mean ± SD	16.61 ± 3.64	13.58 ± 2.01	**0.003 ^b^**
	Median (min–max)	16.82 (10.47–23.76)	13.95 (10.38–16.55)	

SD: standard deviation, Min: minimum, Max: maximum, ^a^: Student *t*-test, ^b^: Mann–Whitney U test.

**Table 7:** Comparing alveolar bone structure variables among groups.

Alveolar Bone Structures			Control Group	Stainless Steel Crown	Pediatric Zirkonia Crown	*p*-Value
Cortical bones (MPa)	Maximum principal stresses	Mean ± SD	9.02 ± 1.85	9.74 ± 2.11	9.16 ± 1.99	0.477 ^a^
		Median (min–max)	8.60 (6.16–13.19)	9.76 (6.36–13.63)	9.25 (6.36–13.24)	
	Minimum principal stresses	Mean ± SD	7.19 ± 1.94	8.08 ± 1.88	7.92 ± 1.98	0.306 ^a^
		Median (min–max)	7.27 (3.48–10.02)	8.07 (4.14–12.43)	8.34 (4.51–10.82)	
Cancellous bone (MPa)	Maximum principal stresses	Mean ± SD	1.22 ± 0.20	1.24 ± 0.22	1.30 ± 0.26	0.493 ^b^
		Median (min–max)	1.23 (0.77–1.58)	1.17 (0.84–1.76)	1.33 (0.75–1.74)	
	Minimum principal stresses	Mean ± SD	4.22 ± 1.10	4.34 ± 1.63	4.22 ± 1.03	0.950 ^a^
		Median (min–max)	4.07 (2.53–6.74)	4.58 (1.91–6.97)	3.96 (2.79–6.12)	

SD: standard deviation, Min: minimum, Max: maximum, ^a^: one-way ANOVA test, ^b^: Kruskal–Wallis H test.

## Data Availability

The datasets used and/or analyzed during this study are available from the corresponding author upon reasonable request.
